# Mitochondria Quality Control and Male Fertility

**DOI:** 10.3390/biology12060827

**Published:** 2023-06-06

**Authors:** José Costa, Patrícia C. Braga, Irene Rebelo, Pedro F. Oliveira, Marco G. Alves

**Affiliations:** 1 Unit for Multidisciplinary Research in Biomedicine (UMIB), Institute of Biomedical Sciences Abel Salazar (ICBAS), University of Porto, 4050-313 Porto, Portugal; jliuze@gmail.com (J.C.); patriciacbraga.1096@gmail.com (P.C.B.); 2 ITR-Laboratory for Integrative and Translational Research in Population Health, 4050-600 Porto, Portugal; 3 Laboratory of Physiology, Department of Imuno-Physiology and Pharmacology, ICBAS-School of Medicine and Biomedical Sciences, University of Porto, 4050-313 Porto, Portugal; 4 UCIBIO-REQUIMTE, Laboratory of Biochemistry, Department of Biologic Sciences, Pharmaceutical Faculty, University of Porto, 4050-313 Porto, Portugal; irebelo@ff.up.pt; 5 Associate Laboratory i4HB-Institute for Health and Bioeconomy, Laboratory of Biochemistry, Department of Biologic Sciences, Pharmaceutical Faculty, University of Porto, 4050-313 Porto, Portugal; 6 LAQV-REQUIMTE, Department of Chemistry, University of Aveiro, 3810-193 Aveiro, Portugal; pfobox@gmail.com

**Keywords:** male fertility, mitochondrial quality control, non-communicable diseases, spermatozoa

## Abstract

**Simple Summary:**

Mitochondria play a crucial role in numerous cellular processes, including energy production, apoptosis, and calcium homeostasis. In the male reproductive system, mitochondria are particularly important for the development and maintenance of germ cells, which ultimately lead to the production of healthy sperm. Dysfunction in mitochondrial physiology can lead to an imbalance in reactive oxygen species, which can have detrimental effects on sperm quality. Thus, mitochondrial quality control can ultimately define male reproductive capacity. Studies have shown that non-communicable diseases such as obesity, diabetes, and cardiovascular disease can have a negative impact on mitochondrial function in sperm, leading to decreased sperm motility, concentration, and viability. Therefore, understanding and managing mitochondrial quality control could be a valuable approach to developing new strategies to combat male infertility. Herein we discuss the relevance of mitochondria quality control to male fertility, particularly the role of oxidative stress and the parameters needed to be evaluated.

**Abstract:**

Mitochondria are pivotal to cellular homeostasis, performing vital functions such as bioenergetics, biosynthesis, and cell signalling. Proper maintenance of these processes is crucial to prevent disease development and ensure optimal cell function. Mitochondrial dynamics, including fission, fusion, biogenesis, mitophagy, and apoptosis, maintain mitochondrial quality control, which is essential for overall cell health. In male reproduction, mitochondria play a pivotal role in germ cell development and any defects in mitochondrial quality can have serious consequences on male fertility. Reactive oxygen species (ROS) also play a crucial role in sperm capacitation, but excessive ROS levels can trigger oxidative damage. Any imbalance between ROS and sperm quality control, caused by non-communicable diseases or environmental factors, can lead to an increase in oxidative stress, cell damage, and apoptosis, which in turn affect sperm concentration, quality, and motility. Therefore, assessing mitochondrial functionality and quality control is essential to gain valuable insights into male infertility. In sum, proper mitochondrial functionality is essential for overall health, and particularly important for male fertility. The assessment of mitochondrial functionality and quality control can provide crucial information for the study and management of male infertility and may lead to the development of new strategies for its management.

## 1. Introduction

Mitochondria, commonly known as the “powerhouses of the cell”, are intricate organelles present in all eukaryotic cells. They consist of two membranes, the outer mitochondrial membrane (OMM) and the inner mitochondrial membrane (IMM), which separate the mitochondrial matrix from the cytoplasm of the cell and define the intermembrane space and mitochondrial matrix [1]. While OMM is highly permeable, IMM is selectively impermeable to most substances. This property is essential for the development of specific transport systems that regulate the movement of molecules to and from mitochondria [1]. The IMM can be further divided into two subdomains, an inner boundary membrane which is in contact with the OMM and the cristae membrane [2]. The cristae membrane is responsible for enclosing the oxidative phosphorylation (OXPHOS) respiratory chain complexes, including complexes I, II, III, and IV [3].

Mitochondria play a critical role in maintaining cellular homeostasis, by facilitating distinct and essential physiological processes such as bioenergetics, biosynthesis, and cell signalling. One of its primary functions is the synthesis of ATP, which occurs through cellular respiration and involves the conversion of ADP and phosphate ions into ATP by the mitochondrial ATP synthase. ATP is the primary energy currency of the cell and is required for various cellular processes, including muscle contraction, protein synthesis, and signal transmission [2]. ATP is also used in a variety of cellular functions, including biosynthesis and degradation of proteins, maintenance of membrane potentials [4] and also to many other physiological functions. Furthermore, mitochondria are known to be a major source of reactive oxygen species (ROS). Specifically, ROS are primarily generated in the respiratory complexes I and III of the electron transport chain (ETC) [5]. Mitochondria regulate the production of ROS through several mechanisms, including the modulation of mitochondrial membrane potential (MMP), the redox state of the ETC complexes, and the availability of oxygen [2,6]. These organelles are considered cell signalling centres and may participate in stress responses [7]. In addition to their primary functions, mitochondria are also involved in several other biological processes. They play a role in aging, thermogenesis, and calcium storage, thereby contributing to the maintenance of cellular calcium homeostasis [2]. Mitochondria use fission and fusion as the primary processes to regulate multiple aspects, including their distribution in the cytoplasm, as well as their size, shape, and number [2]. Mitochondrial quality control is maintained through various processes, including mitochondrial fission and fusion, biogenesis, mitophagy and apoptosis [8]. All these processes are essential for several physiological functions, including male reproduction.

Mitochondria play a crucial role in the male reproductive tract, contributing to the occurrence of spermatogenesis (sperm production) and oocyte fertilization. Spermatogenesis occurs in the seminiferous tubules, where germ cell proliferation and differentiation processes lead to the production of a high quantity of spermatozoa, which heavily rely on mitochondrial function [9]. It can be divided into three phases—spermatogonia mitosis (amplification of spermatogonial stem cells (SSCs)), spermatocytes meiosis (reduction in the chromosome number), and spermiogenesis where the round shaped spermatids are transformed into a more elongated shape [10]. Mitochondria found in spermatogonia and early spermatocytes are small and conventional, exhibiting limited OXPHOS activity. As spermatogenesis progresses to later stages, such as spermatocytes, spermatids, and spermatozoa, the mitochondria undergo a transformation to become more condensed, elongated, and efficient in their OXPHOS activity [11].

**Figure 1:**
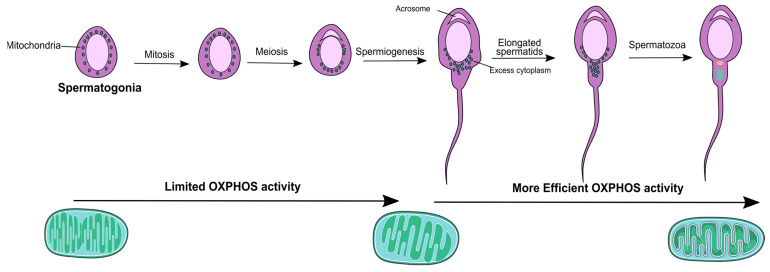
Schematic representation of the stages of spermatogenesis and the corresponding alterations in mitochondrial morphology. Mitochondria has a more orthodox shape in the early stages and become increasingly elongated and condensed as spermatogenesis progresses exhibiting a more efficient Oxidative Phosphorylation (OXPHOS) activity.

Changes in mitochondria morphology depicted in Figure 1 appear to occur prior to their structural association with the sperm flagellum. While most mitochondria are lost during spermiogenesis, some are arranged within the developing spermatid tail [12] and are metabolically more efficient [13] (Figure 1).

In testicular tissue, mitochondria have multiple functions that include energy production [14], steroid hormones production in the testis [15], the maintenance of cell proliferation [16] and cell death [17]. Moreover, in spermatozoa, mitochondria-generated ROS play a vital role in the physiological processes that enable these cells to fertilize an oocyte. These processes include biochemical changes linked to tyrosine phosphorylation, cholesterol release, and the interaction between sperm and egg. However, excessive ROS production can lead to oxidative stress (OS) and contribute to several deleterious events [18]. Human spermatozoa contain only one copy of mtDNA per mitochondrion [19] and their mtDNA sequence is identical to that found in somatic cells. However, the absence or weakness of DNA repair mechanisms in sperm mtDNA leads to a higher mutation rate [2]. Mitochondria in spermatozoa play a crucial role in ATP production, which is essential for promoting sperm motility [20], thus human spermatozoa motility depends on the correct performance of the OXPHOS [21]. Overall, the appropriate functionality of the ETC is inexorable to mitochondrial performance which, in turn, is associated with sperm function [2]. Any alterations that affect mitochondrial quality and performance in sperm have the potential to cause male infertility. Therefore, this review aims to explore the role of mitochondria throughout the male reproductive tract and how their quality control, including dysfunction in response to various metabolic cues such as metabolic disorders and environmental pollutants, may affect male fertility.

## 2. Mitochondria Physiology throughout the Male Reproductive Tract

As stated, spermatogenesis is an event that can be divided in three phases to transform spermatogonial stem cells (SSCs) into spermatozoa and occurs in the seminiferous tubules of the testis [13]. In the spermatozoa, there are between 70–80 mitochondria located in the midpiece [12,13]. Notably, there are metabolic and morphological changes in mitochondria physiology along the different cellular microenvironment of the testis which encloses SSCs, spermatocytes and spermatids [13]. Sertoli cells play a vital role in maintaining the homeostasis of the testis by forming the blood–testis barrier (BTB), which creates a specialized microenvironment in the adluminal space of the seminiferous epithelium [22]. The BTB is a highly specialized tight junction located in the adluminal compartment of the seminiferous epithelium. Its main function is to prevent autoantigens present in spermatogenic cells from being recognized by immune cells of the host [22,23]. The SSCs are located at the base of the BTB, which enables them to utilize nutrients and glucose from the blood supply to produce ATP [24]. Mitochondria in SSCs are immature and highly vacuolated, with a spherical shape and a low electron-lucid matrix and few cristae. In contrast, spermatocytes and spermatids possess a large number of mature mitochondria [25]. Throughout spermatogenesis, germ cells go through several microenvironments with varying concentrations of glucose and metabolites as SSCs are turned into mature sperm [26]. During this process, the mitochondria not only increase in number but also change shape, particularly in meiotic prophase I [26]. These variations in mitochondrial size and shape depend on the proper functioning of fusion/fission and mitophagy, which are essential for maintaining intracellular homeostasis [27], as it will be explained further in detail. Moreover, as spermatogonia and SSCs are located in the basal compartment, they have direct contact with blood and interstitial fluid, providing them with access to glucose and other metabolites. However, spermatocytes and spermatids have limited access to these nutrients [26]. The differential access to metabolites between the basal compartment and adluminal compartment reflects the changing metabolic needs of male germ cells during the various stages of spermatogenesis. It appears that spermatogonia primarily rely on glucose to produce energy through glycolysis [28], while spermatocytes and spermatids rely on lactate provided by Sertoli cells, as well as pyruvate, as their main energy sources [29]. The lactate produced by Sertoli cells is converted to pyruvate by lactate dehydrogenase. Pyruvate is then utilized in mitochondria to produce ATP, indicating that spermatocytes and spermatids in the adluminal compartment are dependent on mitochondrial OXPHOS activity for energy generation [26]. The high energy demand of meiotic spermatocytes, particularly during the meiotic prophase I (MPI), requires a substantial supply of lactate and pyruvate. This demand is expected given the significant energy needed to complete meiosis, including DNA replication, recombination, and chromosomal segregation [26]. This phase represents approximately 90% of meiosis, a quarter of the spermatogenic process, and can be further divided into four different stages (leptotene, zygotene, pachytene, and diplotene) [30]. Electron microscopy studies conducted in rodent testes have shown that there is an increase in OXPHOS activity during the MPI stage of meiosis. Specifically, during pachytene, mitochondria present in the spermatocytes assume a more elongated shape while the cristae are more compact [31]. This type of mitochondrial organization has been shown to be associated with increased OXPHOS activity [32]. Conversely, in spermatids that have completed the meiosis process, the mitochondria are fragmented, and the cristae do not have either an orthodox or a condensed shape, indicating a return to reliance on glycolysis [31].

Sertoli cells play a crucial role in regulating the number of germ cells in the testis. They do this by modulating apoptosis during mitosis, which prevents the overproduction of germ cells and maintains the homeostasis of the testis [33]. After phagocytosis of apoptotic spermatogenic cells, the lipids from these cells are degraded into fatty acids which undergo β-oxidation to generate ATP in Sertoli cells. The degradation of the apoptotic cells into lipids results in a significant increase in long-chain acyl-CoA dehydrogenase, an enzyme involved in β-oxidation, in the mitochondria of Sertoli cells. This process of utilizing lipids from apoptotic germ cells for energy production is an important mechanism for maintaining the metabolic activity of Sertoli cells and for supporting the development of germ cells [34]. In addition to promoting β-oxidation of lipids, Sertoli cells also play a role in regulating mitochondrial biogenesis and OXPHOS activity in germ cells. Activin A, a protein secreted by Sertoli cells, has been shown to promote mitochondrial biogenesis and the formation of elongated cristae-rich mitochondria in spermatocytes and spermatids. This is achieved through transcriptional regulation of key genes involved in mitochondrial biogenesis, including peroxisome proliferator-activated receptor-γ coactivator 1α, nuclear respiratory factor (NRF) 1, and NRF2 [24,31]. These transcription factors activate the expression of genes involved in mitochondrial DNA replication, transcription, and translation, as well as those encoding components of the electron transport chain, leading to increased OXPHOS activity in germ cell [35].

Leydig cells are another crucial somatic cell type found in the testis. Their role in male germ cell differentiation is less well studied than that of Sertoli cells [13]. Leydig cells are in the interstitial tissue of the testis and are responsible for the production and secretion of steroid hormones, particularly androgens such as testosterone. These hormones are synthesized and released into the bloodstream in response to the stimulation of luteinizing hormone (LH) from the pituitary gland [36]. While the exact role of Leydig cells in male germ cell differentiation is not fully understood, it is known that androgens play an important role in the regulation of spermatogenesis and male fertility. Testosterone plays an essential role in spermatogenesis as it is required for the development of spermatogonia and the initiation of meiosis. It also regulates the release of spermatids from Sertoli cells into the lumen of the seminiferous tubules [37]. When circulating LH binds to its receptor on Leydig cells, it triggers a signalling cascade that increases the production of cyclic AMP (cAMP). This increase in cAMP promotes the transport of cholesterol to the inner mitochondrial membrane (IMM) and initiates the first step of testosterone production. This step involves the conversion of cholesterol into pregnenolone by the P450 cholesterol side-chain cleavage enzyme (P450scc). Pregnenolone then undergoes further enzymatic reactions to eventually produce testosterone [38]. When referring to cholesterol in mitochondria, its typical functions are biogenesis, membrane maintenance and the production of steroids [39]. Although the primary function of mitochondria is to produce ATP, this is not the case for Leydig cells. The morphology of the cristae in Leydig cells does not allow the connection between the F1 complexes of ATP synthase in the mitochondrial matrix when they are close to the mitochondrial membrane [40]. Therefore, Leydig cells produce less ATP, and their mitochondria are mainly responsible for the production of steroid hormones, such as testosterone, through the steroidogenesis pathway.

After leaving the testicular seminiferous tubules, spermatozoa have to mature in the epididymis where they gain the capacity to reach and fertilize the oocyte [41]. Epididymal cells create a unique microenvironment in the epididymal lumen to support sperm maturation. This microenvironment is maintained by epithelial cells, which keep the pH acidic and the bicarbonate concentration low. This ensures that the spermatozoa do not become prematurely activated while they are maturing in the epididymis [41]. The acidic pH is supported by proton secretion by V-ATPases, which are abundant in the apical membrane and intracellular vesicles of the narrow and clear cells in the epididymis [41]. The narrow and clear cells present in the epididymis are considered mitochondria-rich cells and these abundant mitochondria are linked to the acidification of the lumen due to their carbonic anhydrase activity, endocytic activity and most importantly, proton secretion through V-ATPase [42].

It is important to mention the importance of ROS in spermatozoa capacitation, since protein tyrosine phosphorylation is regulated by ROS production [43]. The presence of ROS triggers a cascade of biochemical reactions that enhance sperm motility. First, ROS induces the conversion of ATP to cAMP by the enzyme adenylyl cyclase. Next, cAMP activates protein kinase A (PKA), which further stimulates ROS production and the enzyme NADPH oxidase. PKA also phosphorylates serine and tyrosine residues, leading to activation of protein tyrosine kinase (PTK). Finally, PTK triggers phosphorylation of tyrosine residues in the sperm flagellum axoneme, resulting in increased motility [43]. Another key event during capacitation is the mobilization of calcium ions, which is also triggered by ROS. This increase in calcium ions leads to the cleavage of PIP2 (phosphatidylinositol-4,5-biphosphate), producing DAG (diacylglycerol). The presence of DAG and PKC (protein kinase C) induces the phosphorylation of phospholipase A2, a membrane enzyme that plays a critical role in sperm function. This phosphorylation event increases the fluidity of the spermatozoa membrane, enabling it to fuse with the oocyte. This membrane fusion is a crucial step in fertilization and requires precise coordination between sperm and oocyte. [44]. Overall, the interplay between ROS, calcium ions, PKC, and phospholipase A2 during capacitation is a finely tuned process that ensures successful fertilization.

Spermatozoa are highly vulnerable to the harmful effects of ROS, which include superoxide anion, hydrogen peroxide, hydroxyl radical, nitric oxide, and peroxynitrite. Elevated levels of ROS can lead to oxidative damage, impairing sperm function [45]. This impairment translates into a loss of sperm motility and mitochondrial activity and also the possibility of losing the ability to fertilize oocytes [46]. Spermatozoa have limited capacity to repair oxidative damage, as their chromatin is highly compacted and lacks the necessary machinery to synthesize new proteins. Therefore, they rely on antioxidant enzymes that are produced during spermatogenesis and epididymal maturation to provide some degree of protection [46]. During epididymal maturation, the plasma membrane of spermatozoa becomes enriched with polyunsaturated fatty acids (PUFAs) [13]. While PUFAs contribute to the integrity of the sperm membrane and improve its ability to fuse with the oocyte during fertilization, they also increase the sperm susceptibility to OS. Therefore, the balance between ROS and antioxidants is critical for maintaining proper sperm function and fertility [47].

As discussed above, to fertilize an oocyte, spermatozoa must be prepared through morphological alterations, such as the remodelling of the plasma membrane. Those changes will assist the penetration of the oocyte and the survival of sperm in the female reproductive tract. This process, known as capacitation, is mainly characterized by the activation of tyrosine kinase via cAMP [13]. Capacitation is related to the improvement of sperm characteristics such as membrane fluidity, motility, and calcium inflow, facilitating spermatozoa to penetrate the oocyte [48]. Glycolysis and mitochondrial OXPHOS are the main metabolic pathways that support capacitation, as ATP is required for this process to occur [49]. In a study by Carrageta and colleagues, human spermatozoa were incubated under in vitro capacitation conditions with varying glucose concentrations. Spermatozoa incubated without glucose exhibited a lower viability, while those incubated with glucose maintained viability over time [49]. The study also revealed that the sperm cells incubated with glucose exhibited higher levels of tyrosine residue phosphorylation, which is a commonly used biomarker for sperm capacitation. This finding demonstrates the importance of glucose in promoting human sperm capacitation [49]. ATP production via OXPHOS occurs in the midpiece of spermatozoa, primarily supporting sperm motility. However, it is also crucial for maintaining chromatin structure and regulating the acrosome reaction in spermatozoa [50]. Zhang and colleagues investigated sperm MMP in young college students, using the N-α-benzoyl-DL-arginine-para-nitroanilide HCl (BAPNA) substrate method to measure acrosin activity and DNA fragmentation index (DFI) to assess chromatin integrity. Their findings indicated that individuals with low MMP had reduced acrosin activity and lower DFI when compared to those with high or moderate MMP [50]. This study also found that sperm MMP dissipation led to ROS overproduction and reduced ATP content [50]. Overall, mitochondria play a vital role throughout the male reproductive tract, and their dysfunction can cause errors during spermatogenesis, sperm capacitation and oocyte fertilization, leading to male infertility.

## 3. Relevance of Mitochondrial (Dys)function to Male Fertility

Mitochondrial activity plays a crucial role in all stages of male reproductive potential, from spermatogenesis to oocyte fecundation. In spermatozoa, mitochondria are located in the midpiece and form the mitochondrial sheath that surrounds the axoneme [51]. This structure is connected to the axoneme by a reticulum of filaments and synthesizes ATP, which is necessary for the proper sperm function [52,53]. Recent comparative studies have shown that the amount of mitochondria in the sheath is directly related to sperm velocity and ATP production. For instance, Gu and colleagues studied sperm morphology and mitochondrial functions in 10 mammalian species, including humans, and found that a higher number of mitochondria in the sheath was positively correlated with increased sperm motility and ATP production [54]. In humans, studies have shown that low sperm motility is associated with smaller midpieces, abnormally assembled mitochondria, and mitochondrial membranes with structural defects [51]. These findings suggest a direct correlation between the correct mitochondrial structure and fertilization rates [55].

Sperm quality is closely linked to proper mitochondrial function, and disruptions in the ETC pathway can adversely affect sperm quality [51]. In humans, the MMP, which serves as an indicator of energy and mitochondrial functions, has been found to be associated with sperm viability [56] and the ability to perform acrosome reaction [57] as well as the capacity to fertilize oocytes naturally or in vitro [58]. When measuring the enzymatic activity of the respiratory chain complexes, Ruiz-Pesini and colleagues used specific modulators of the ETC complexes and found that their correct functioning influenced sperm parameters such as motility and vitality [21]. They were also able to demonstrate a direct correlation between mitochondrial enrichment of complex II and more efficient spermatogenesis, leading to a higher number of ejaculated spermatozoa [21]. Lastly, mitochondrial respiration and oxygen consumption have been linked to higher sperm motility and capacitation [59], further highlighting the direct relationship between mitochondrial function and healthy sperm physiology, as we summarized in Table 1.

**Figure 2:**
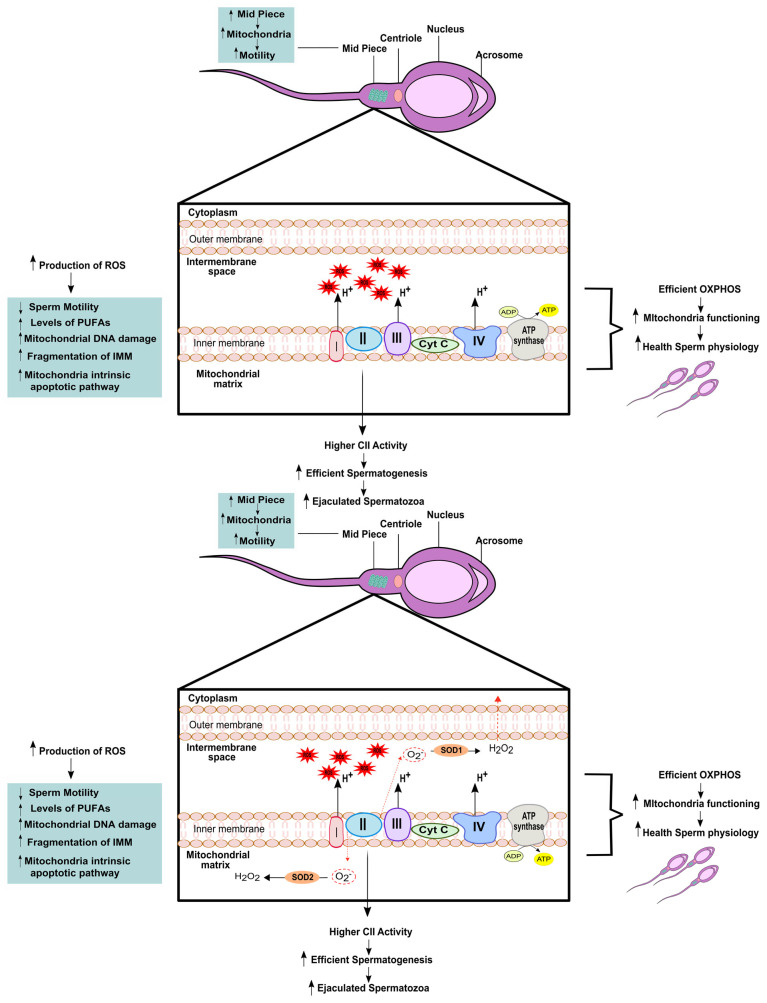
Schematic summary of the complex relationship between mitochondrial quality and spermatozoa health. An efficient oxidative phosphorylation (OXPHOS), particularly at the complex II (CII) level in the electron transport chain, will lead to more ejaculated spermatozoa presenting a better health sperm physiology. Furthermore, a higher mid piece of the spermatozoon is directly linked with a higher motility. However, an imbalance between reactive oxygen species (ROS), will lead to lower sperm motility as well as an increase in mitochondrial DNA damage, an increase in polyunsaturated fatty acids (PUFAs) and increase in the mitochondrial apoptotic pathway. The enzymes superoxide dismutase 1 and 2 (SOD1 and SOD2, respectively) composed the metabolic machinery that handles reactive oxygen species (ROS) in the mitochondrial matrix. Specifically, these enzymes are able to convert superoxide radical anion (O_2_^•−^) into hydrogen peroxide (H_2_O_2_).

Mitochondria are also involved in other processes that can affect sperm quality, such as generation of ROS, calcium control and cell signalling [51] (Figure 2). The overproduction of ROS by leukocytes, immature germ cells, and defective spermatozoa can exceed the physiological threshold leading to oxidative stress, which is very deleterious for the sperm [51]. Therefore, it is crucial to maintain a delicate balance of ROS levels in the male reproductive system. Oxidative stress can have a negative impact on sperm quality and function, including reducing sperm motility, affecting mitochondrial activity, and ultimately, reducing the ability of sperm to successfully fertilize the oocyte [60]. The high levels of PUFAs in the sperm membrane make them highly susceptible to lipid peroxidation caused by ROS. This peroxidation of lipids can also trigger the generation of ROS by sperm mitochondria. PUFAs penetrate the IMM and inhibit the proper flow of electrons in the ETC, leading to the production of superoxide anions, oxidative DNA damage [61] and DNA base oxidation [62]. When OS is present, there is increased amount of ROS that stimulates more generation of ROS [2]. This is due to the formation of aldehyde–protein adducts, which are by-products of lipid peroxidation and bind to proteins in the mitochondrial ETC, causing the generation of more ROS in the mitochondria [63] (Figure 2). This perpetuates a cycle of ROS generation and the resulting OS, which ultimately leads to apoptosis of spermatozoa. This occurs because sperm mitochondria are extremely sensitive to oxidative stress, despite being the main intracellular ROS generator [51]. Therefore, the maintenance of ROS levels within the physiological range is crucial for preserving sperm quality and function.

Koppers and colleagues demonstrated that mitochondrial ROS generated from complex III can cause the release of hydrogen peroxide into the extracellular space without detectable peroxidative damage. In contrast, the induction of mitochondrial ROS from complex I results in leakage into the mitochondrial matrix, leading to peroxidative damage to the IMM [61]. This damage to the IMM caused by mitochondrial ROS from complex I may result in the opening of the mitochondrial permeability transition pores and fragmentation of the IMM, leading to activation of the mitochondrial intrinsic apoptotic pathway [64]. Since the chemical triggers for apoptosis that exist in somatic cells are not present in the spermatozoa, these will automatically undergo truncated apoptosis unless pro-survival factors are able to prevent this process [65]. However, the specific signalling pathways that mediate these processes in spermatozoa are not well understood. An important signalling molecule is calcium, which plays a crucial role in sperm function. Calcium is a messenger that participates in a variety of cellular processes. In humans, it has been demonstrated to be crucial in sperm function, being involved in the movement of the flagellum, capacitation, acrosome reaction and chemotaxis [66]. Calcium-dependent pores in the sperm IMM open in response to high intracellular calcium levels, allowing calcium to enter mitochondria and reduce MMP [67], activating the apoptotic pathway [51].

Research on sperm apoptosis has demonstrated that apoptotic markers in sperm are similar to those found in somatic cells. In both cases, phosphatidylserine is externalized in the plasma membrane, mitochondrial integrity is compromised, caspase is activated, and DNA damage occurs [68,69,70,71]. Despite the similarities, understanding and explaining the apoptotic process in spermatozoa remains a challenge due to the unique physiological conditions of these cells [51]. Apoptotic markers of immature sperm, such as blebbing of the plasma membrane, formation of apoptotic bodies, impaired mitochondrial integrity, defects of the nuclear envelope, and fragmentation of the nucleus, have been associated with infertility in men with reproductive issues [72]. Conversely, these markers are not present in fertile men and mature sperm [73]. The mitochondria, responsible for the intrinsic apoptotic pathway, are thought to play a crucial role in triggering apoptosis in spermatozoa. For example, a study conducted on infertile men with spermatic alterations showed a direct positive correlation among ROS, cytochrome c outside the mitochondria, and the induction of caspases 9 and 3. In another study by Paasch and collaborators it was shown that inducing apoptosis in human sperm leads to higher activity of caspase 9 and 3, along with lower MMP and sperm motility [74]. Activation of caspases 9 and 3 is associated with low sperm quality, higher DNA fragmentation, and reduced fertilization capabilities. Furthermore, when caspase 3 is activated together with disrupted MMP, it leads to the release of phosphatidylserine in spermatozoa [75]. These findings are consistent with the notion that the intrinsic apoptotic pathway involving mitochondria plays a role in regulating sperm apoptosis.

The phosphatidylinositol 3-kinase (PI3K)/AKT signalling pathway plays a crucial role in regulating cell cycle progression, growth, proliferation, survival, and migration [2]. Under stress, the activation of AKT promotes cell survival [76]. Activation of the PI3K enzyme leads to the phosphorylation of AKT1, which, in turn, silences apoptotic pathway promoters, helping to maintain the functionality of spermatozoa. [65]. Inhibition of the PI3K enzyme in this pathway results in the dephosphorylation of AKT1, leading to the initiation of the intrinsic apoptotic pathway in spermatozoa [2]. Consequently, this leads to caspase activation, increased production of mitochondrial ROS, oxidative DNA damage, and reduced sperm motility [77]. Due to the unique architecture of the sperm head, which separates the nucleus from the mitochondria and cytoplasm in the sperm midpiece, endonucleases that are activated during apoptosis are unable to cleave nuclear DNA. As a result, DNA fragmentation in nuclear DNA is not a consequence of apoptosis [77].

Multiple changes can occur in mtDNA at the molecular level, including deletions, substitutions, and other point mutations. These changes can result in poor sperm quality and subsequently male infertility [14]. Mouse models have been used to study the role of OXPHOS in spermatogenesis. Specifically, mice with error-prone mtDNA replication and a mutation in the subunit of the mtDNA polymerase γ have been found to have infertility issues [78]. The “mtDNA mutator” mice presented early degradation in multiple organ systems, particularly the testes, where severely degenerated seminiferous tubes and germ cell depletion were observed at 10 months of age [78]. Jiang and colleagues demonstrated that altering the expression levels of the mtDNA regulator, mitochondrial transcription factor A (Tfam), could affect infertility in mtDNA mutant mice. Increasing Tfam expression levels mitigated the infertility phenotype, while decreasing levels worsened the phenotype [79]. Additionally, mitochondrial dysfunction has been shown to affect MPI in mice. Mice with a high level (4696 bp) pathogenic deletion in mtDNA had low OXPHOS activity, which resulted in meiotic arrests at the transition between zygotene and pachytene [15]. In mice lacking the testis-specific adenine nucleotide translocator 4 (Ant4), a genetic ablation caused spermatogenic arrest in the leptotene phase of MPI [80].

In humans, it has been reported that males with poor sperm quality have a higher prevalence of sperm mitochondrial DNA deletions compared to normozoospermic males [81]. Large-scale mtDNA deletions have been reported to occur more frequently in males with obstructive azoospermia compared to fertile and infertile men with non-obstructive azoospermia [2]. This can be attributed to blockage in the reproductive tract, which creates a higher level of OS and mitochondrial dysfunction. These deletions can affect the OXPHOS system, causing a decrease in ATP production, which can lead to poor sperm motility and decreased fertilization capacity. In addition, these deletions can also cause oxidative damage to sperm DNA, leading to increased levels of sperm DNA fragmentation and subsequent infertility. Additionally, large deletions or a high number of deletions can lead to disruption of ETC, reducing the efficiency of oxidative phosphorylation and impairing ATP synthesis [82]. This mitochondrial dysfunction can affect sperm quality and motility. Indeed, one of the most sensitive biomarkers of male fertility is sperm mtDNA copy number [83]. When the copy number of human mtDNA is altered, both sperm motility and fertilization capacity are be affected [84]. It has been demonstrated that infertile males or those with abnormal semen parameters have a decline in mtDNA integrity when copy number increases [85]. Conversely, a decrease in mtDNA copy number in spermatozoa has been associated with lower sperm motility [86]. The regulation of proteins in sperm has been shown to be differentially affected in patients with decreased sperm motility [87], particularly proteins involved in the fibrous sheath and energy production [88], as well as those involved in spermatogenesis, sperm maturation, sperm tail structure and motility, and mitochondrial quality control [89]. In samples from patients with reduced sperm motility, it has been shown differential protein expression of several proteins. Proteins involved in energy and metabolism, such as triose-phosphate isomerase, glycerol kinase 2, and succinyl-CoA:3-ketoacid co-enzyme A transferase 1, are expressed at higher levels compared to those involved in sperm motility and structure, as well as stress response [90]. This suggests a possible shift in energy production towards glycolysis rather than OXPHOS, which may be a compensatory mechanism to maintain ATP levels and reduce OS in spermatozoa with impaired motility. Additionally, proteins involved in mitochondrial quality control, such as Lon peptidase 1 and prohibitin, were downregulated, suggesting a possible role of mitochondrial dysfunction in reduced sperm motility. In many cases, mitochondrial dysfunction leading to errors in spermatogenesis, sperm capacitation, and oocyte fertilization, which affect sperm quality, motility, and fertilization capacity, are caused by external factors such as metabolic diseases. Thus, a deeper understanding of the impact of metabolic diseases on mitochondrial quality control is imperative to manage and ensure adequate spermatogenesis and prevent male infertility. Appropriate management of metabolic diseases may include lifestyle modifications, pharmacological interventions, or other treatments aimed at reducing mitochondrial dysfunction and improving overall metabolic health. By doing so, we can improve male fertility outcomes and reduce the incidence of infertility caused by metabolic diseases.

**Table 1:** Spermatozoa fitness alterations due to mitochondrial dysfunction.

Mitochondrial Associated Mechanism	Spermatozoa Outcomes	References
↓ MMP	↓ sperm viability ↓ Acrosome reaction	[57]
↓ ETC	↓ sperm motility ↓ sperm vitality	[21]
↑ amount of mitochondria in the sheath	↑ sperm morphology ↑ ATP production	[53]
Enrichment of complex II	More efficient spermatogenesis ↑ ejaculated spermatozoa	[21]
↑ Oxidative stress	↓ sperm motility ↓ fertilization capacity	[59]
Damage in IMM	↑ apoptotic pathway ↓ MMP ↓ sperm motility	[63]
↓ mtDNA integrity	Poor sperm quality	[14,83,85]

## 4. Non-Communicable Diseases and Environmental Impact on Mitochondrial Quality of Testicular Cells and Sperm

When analysing mitochondrial quality in male fertility, it is crucial to take into account both internal mitochondrial parameters and cues that may alter the mitochondria’s proper operation. Indeed, non-communicable diseases (NCDs) are an example of a condition that can impact male fertility by altering mitochondrial function. NCDs refer to chronic, non-infectious and non-transmissible diseases that can have a significant impact on overall health and well-being [91]. By studying the relationship between NCDs and mitochondrial function, we can gain a deeper understanding of how these diseases can affect male fertility and develop targeted strategies to mitigate their negative effects. Among the most common NCDs are cardiovascular and neurological diseases, as well as cancer and diabetes. Obesity, which is associated with a group of other NCDs including diabetes mellitus (DM), hypertension, metabolic syndrome, non-alcoholic fatty liver disease, and cardiovascular diseases, is also a major risk factor [91]. In addition, recent studies have linked obesity to some types of cancer such as colorectal, liver, and prostate [92].

**Figure 3:**
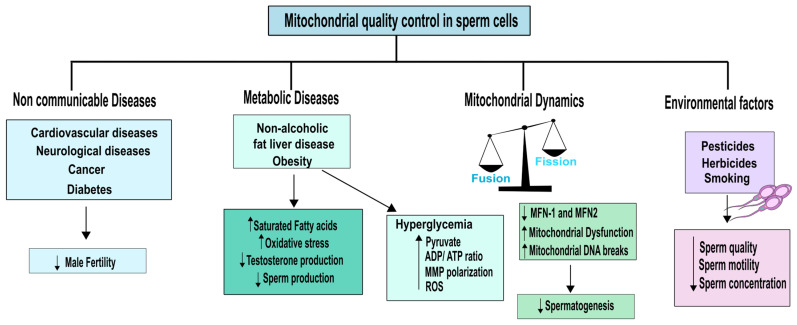
Overview of the interconnected homeodynamics of sperm cells and mitochondrial quality control. Various factors can influence this delicate balance, including non-communicable diseases, metabolic diseases, mitochondrial dynamics, and environmental factors. An imbalance in any of these conditions can result in unhealthy spermatozoa, compromising male fertility. Abbreviations: ROS- Reactive oxygen species; MMP—mitochondrial membrane potential; MFN-1—mitofusin 1 and MFN-2—mitofusin 2.

Obesity is associated with mitochondrial dysfunction, which results in lower energy metabolism. When nutrient supply is abundant, cells produce more mitochondria. However, if nutrient supply remains excessive, the mitochondrial system becomes overloaded, leading to dysfunction and accumulation of non-oxidized lipid products. This causes accumulation of fat and OS, which leads to mitochondrial damage and a further decline in energy metabolism [91]. Excess adipose tissue is known to increase aromatase activity, which converts testosterone to oestradiol, leading to decreased testosterone levels in men [93]. Since testosterone is crucial for regulating spermatogenesis, this reduction in testosterone levels can significantly reduce sperm production [94]. Moreover, decreased testosterone levels are also accompanied by mitochondrial dysfunction in Leydig cells, resulting in oxidative damage to lipids, proteins, and mtDNA, promoting the production of ROS and reduced ATP levels [95]. Obesity is often correlated with a diet rich in saturated fats (SFA) and low in PUFAs [96]. In humans, a direct correlation between increased amounts of SFA and decreased sperm count and concentration has been described [97]. Conversely, a higher intake of omega-3 PUFAs has been linked to better sperm quality. These findings highlight the importance of a balanced and healthy diet in maintaining mitochondrial function and male fertility, as described in Figure 3.

Regarding PUFAs, it has been suggested to play a role in modulating sperm bioenergetic pathways. Indeed, PUFAs are important components of cell membranes and play a role in regulating mitochondrial function, oxidative stress, and inflammation [98]. There is a isoenzyme form of sperm lactate dehydrogenase (LDH-C4) that is crucial for the nutritional regulation of omega-3 PUFA [98]. This enzyme is present in both the mitochondrial matrix and the cytosol of spermatozoa, and plays a crucial role in the energy metabolism of spermatozoa by catalysing the conversion of pyruvate to lactate and the adjuvant oxidation of NADH [20,99]. LDH-C4 also enables the concurrent advancement of OXPHOS and glycolysis transporting reducing equivalents from the cytosol into the mitochondria and by regenerating NAD+, respectively [94]. It has also shown that a that a diet rich in omega-3 PUFAs can reduce OS in sperm cells by increasing the ratio of aconitase to fumarase activity [98]. The activity ratio of these two Krebs cycle enzymes is considered a marker for mitochondrial production of ROS [100]. On the other hand, a diet high in SFA and low in PUFA has been linked to decreased activity of LDH-C4, pyruvate dehydrogenase, and respiratory enzymes [100].

Despite growing interest in the relationship between diet and reproductive health, the potential effects of dietary carbohydrates on sperm quality remain largely unexplored by scientific research [94]. Glucose is the main fuel for glycolysis in sperm cells, where it is metabolized to pyruvate and/or lactate to produce ATP, the essential energy source for sperm motility. Thus, any reduction in glucose uptake and metabolism by sperm can lead to a decrease in ATP levels, impairing their ability to swim and fertilize an egg [94]. Elevated blood glucose levels have been associated with decreased testosterone production and increased OS in the body [101]. These factors can negatively impact sperm health by impairing mitochondrial function, which is crucial for generating energy and maintaining motility [102]. As a result, high blood glucose levels may lead to reduced sperm motility, highlighting the importance of controlling blood glucose levels for optimal reproductive health. Diabetes mellitus (DM) refers to a group of metabolic disorders characterized by chronic hyperglycaemia resulting from defects in insulin secretion, insulin action, or both. It is a complex and heterogeneous condition, with multiple subtypes and underlying mechanisms, but chronic hyperglycaemia is the defining feature of all forms of DM [103]. The pathogenesis of DM involves a complex interplay of various factors, including an imbalance between free radical formation and the antioxidant defence mechanisms in the body. This OS can result in damage to cellular components, leading to impaired insulin signalling and glucose metabolism [104]. Hence, the dysregulation of the redox balance, characterized by increased free radical formation and/or decreased antioxidant defences, plays a crucial role in the development and progression of DM. DM is associated with a range of vascular and organ-related complications that arise due to overproduction of ROS induced by hyperglycaemia. ROS can cause oxidative damage to various tissues and organs, leading to inflammation, cell injury, and impaired organ function [105]. Mitochondrial dysfunction is a critical factor in the pathogenesis of diabetes, as evidenced by the lower rates of ATP synthesis observed in individuals with a family history of the disease, even before the onset of poor glucose tolerance [106]. Hyperglycaemia promotes the synthesis of pyruvate and increases the flow of reducing equivalents into the ETC [105], resulting in a higher increased ATP/ADP ratio and MMP polarization. However, the significant electrochemical potential difference created by the proton gradient can partially inhibit complex III of the ETC, leading to coenzyme Q accumulation in its reduced form and subsequent generation of superoxide [106]. This increased reduction in coenzyme Q and the resulting production of ROS is suggested to be responsible for mitochondrial dysfunction, which is a key factor in the metabolic abnormalities and tissue histopathology associated with DM [106]. Normally, mitochondria maintain a slightly reduced MMP that generates less ROS, but in hyperglycaemia, hyperpolarization of the MMP leads to increased production of ROS [105].

It was found that exposure to high glucose concentrations causes rapid fragmentation of mitochondria, leading to increased production of ROS [107]. Incubating sperm with high glucose concentrations prevented periodic fluctuations in ROS production by inhibiting mitochondrial fission [107]. This suggests that hyperglycaemic conditions induce dynamic changes in mitochondrial morphology that contribute to the overproduction of ROS. Thus, the mitochondrial fission/fusion machinery represents a potential target for monitoring and regulating acute and chronic ROS production in hyperglycaemia-related disorders [105]. Taken together, these findings indicate that one of the main complications of obesity and DM is the overproduction of ROS and the resulting OS. Understanding the mechanisms underlying ROS production and regulation is critical for developing effective interventions to mitigate the negative consequences of hyperglycaemia and OS. As discussed above, both spermatozoa and germ cells are vulnerable to oxidative stress (OS), which can negatively impact sperm quality by reducing sperm count, motility, and increasing the incidence of abnormalities [108]. Furthermore, oxidative damage to mitochondrial DNA can increase the frequency of large-scale deletions and DNA strand breaks, accelerating germ cell apoptosis and reducing sperm quantity, which is associated with male infertility and lower semen quality [86]. The importance of mitochondrial respiratory activity in mammalian spermatogenesis has also been highlighted by studies linking defects in mitochondrial respiration to meiotic arrest and abnormal sperm morphology [15]. These findings emphasize the crucial role of mitochondrial function and OS regulation in maintaining sperm quality and male reproductive health.

In recent years, there has been an increase in life expectancy, leading to an increase in the age of parents and a corresponding rise in infertility issues. However, there is still limited research on the effects of aging on male fertility and how advanced paternal age affects offspring [109]. Aging in males is associated with changes in the hypothalamic–pituitary–gonadal axis and alterations in the testis, penis, and prostate [12]. Testosterone levels decrease, sperm motility slows, and erectile dysfunction becomes more common in older men [12]. In addition, there is an increase in chromosomal defects and DNA damage, which may have consequences for the offspring [110]. In cells, mitochondrial dysfunction also has a direct correlation with age, where respiratory chain function is affected with advancing age [12]. Studies in mice expressing defective polymerase gamma (POLG), which is essential in mitochondrial DNA replication, have shown premature aging [111]. Using nuclear magnetic resonance (NMR) spectroscopy, Jarak and colleagues were able to identify metabolic changes associated with different stages of reproductive maturity in mice. A notable finding was a significant decrease in testicular creatine content in older mice, indicating changes in the conditions required for male germ cell development [112]. Creatine is a crucial component in the energy metabolism of tissues with high energy demands, including the testis, and assists in ATP replenishment. Therefore, alterations in creatine levels may suggest metabolic changes associated with aging in the testis. In addition, the study showed that advanced age in mice was associated with increased levels of complex I protein, which is linked to ROS overproduction, as well as increased expression levels in the other OXPHOS complexes [112]. However, when comparing 24-month-old mice to 12-month-old mice, there was a significant decrease in the expression levels of the OXPHOS complexes with age, suggesting a decline in mitochondrial function [112].

While NCDs and aging can contribute to mitochondrial dysfunction in the male reproductive tract, there are several environmental factors that can also impact male fertility. In particular, there is growing interest in the potential impact of toxicants, such as herbicides on sperm quality [113]. Despite increased research in this area, our understanding of how these chemicals affect the molecular processes that influence sperm quality remains limited [114]. Anifandis and colleagues demonstrated the negative impact of herbicides on human sperm motility and mitochondrial function by treating spermatozoa with 1 mg/L of Roundup, whose primary active component is glyphosate (GLY) [113]. Using mitochondrial staining, the researchers observed a decrease in sperm motility and mitochondrial staining after one hour of exposure to Roundup, compared to control cells. This suggests that GLY may cause reduced sperm motility by inducing oxidative stress in mitochondria and increasing the production of mitochondrial apoptotic signals [113]. In a more recent study, Ferramosca and colleagues investigated the effects of GLY and glufosinate ammonium (GA) on the efficacy of mitochondrial respiration in human sperm mitochondria. Their findings showed that GLY significantly reduces mitochondrial functionality by lowering oxygen in both the active and passive stages of mitochondrial respiration. In addition, GA may induce mitochondrial permeability by altering the PI3K/AKT complex phosphorylation status, resulting in the loss of motility in human sperm mitochondria [114]. These examples underscore the impact of environmental factors such as pesticides and herbicides on mitochondrial quality control in sperm, which can compromise male fertility.

Cigarette smoking is one of the major health concerns in people of fertile age, and nicotine is its main component. [115]. Detection of nicotine and its major metabolite, cotinine, in the seminal plasma of smokers has demonstrated that tobacco chemicals can penetrate the blood–testis barrier and cause harm to spermatozoa [116]. Components of cigarette smoke are known to be toxic and their intake can lead to testicular microcirculation, DNA, and chromosomal damage in germ cells [117]. Numerous studies have shown that cigarette smoking reduces semen volume, sperm concentration, motility, and normal physiology [118,119,120], while also decreasing the sperm’s ability to fertilize [121,122]. Additionally, sperm from smokers have higher levels of oxidative DNA damage and aneuploidy compared to non-smokers [123]. Chohan and Badawy used phosphorescent analysis to measure oxygen concentrations in sperm suspensions and compared sperm respiration rates in smokers and non-smokers. Their findings revealed that cigarette smoking significantly influences sperm respiration by reducing mitochondrial oxygen consumption [115]. Overall, cigarette smoking has a detrimental effect on male fertility, and its negative impact on sperm motility, morphology, and DNA integrity is well-established. It is important for individuals of reproductive age to avoid cigarette smoking to protect their reproductive health.

While the health advantages of physical activity have been extensively studied for a variety of medical conditions, the impact of exercise on male fertility remains unclear [95]. Several studies indicate that the type, duration, and intensity of exercise have varying impacts on male fertility [95]. Vigorous exercise has been found to reduce male reproductive capacity [124], whereas aerobic, resistance, or combined exercises have been shown to improve male fertility [125]. Although the effects of physical exercise on sperm quality are well established, the underlying mechanisms remain unclear [95]. However, some compelling evidence suggests that high-intensity exercise can induce OS [126,127], which may play a role on impacting male fertility. Aerobic exercise leads to increased oxygen consumption, which is associated with a higher rate of electrons passing through the mitochondrial respiratory chain complexes, possibly causing OS [128,129]. Additionally, catecholamines released during exercise, prostanoid metabolism, xanthine oxidase, and NAD(P)H oxidase are sources of ROS [95]. Regular exercise or chronic anaerobic training can enhance endogenous antioxidant defence mechanisms, reducing oxidative damage [129,130]. However, it has been suggested that rigorous training or prolonged competition periods may increase OS [131]. This is because ROS release can dysregulate the inflammatory and neuroendocrine systems, which may exceed the capacity of the antioxidant system to protect against damage [132]. In healthy young adults who do not exercise regularly, any type of exercise can increase free testosterone concentrations. However, for athletes, only high-intensity exercise appears to lead to an increase in testosterone levels [95]. Recent studies among elite athletes have revealed that their testosterone levels are significantly lower than the physiologically normal range, which could be a consequence of chronic exposure to high levels of aerobic exercise volume and intensity [133,134]. This puts athletes at risk of overtraining and persistent fatigue [95]. Unfortunately, low testosterone levels have been associated with OS, which can result in a decline in sperm quality, thus affecting male fertility [18].

## 5. Mitochondria Quality Control Parameters Essential to Evaluate

Mitochondria play a crucial role in male fertility, contributing to spermiogenesis, capacitation of the spermatozoa, and oocyte fecundation [13]. Therefore, when assessing the quality of mitochondrial functions as a biomarker or sentinel for sperm quality, several factors should be considered along the male reproductive tract. As already explored, mitochondria morphology differs significantly along spermatogenesis. Specifically, mitochondria in SSCs are heavily vacuolated, spherical, and lack cristae. Furthermore, fusion of OMM and IMM is regulated by proteins anchored to the membrane, including mitofusin (MFN)-1,2 and optic atrophy (OPA)-1. These proteins facilitate the fusion of the OMM and IMM, and when they are non-functional, they can lead to the disintegration of the mitochondria [135]. On the other hand, fission is controlled by proteins such as cytosolic dynamin, Fission1 protein (Fis-1), mitochondrial fission factor (MFF), and dynamin-related protein 1 (Drp1). Drp1 is transported from the cytosol to the mitochondria, and a Drp1 deficiency can result in mitochondria hyperfusion [136]. A recent study conducted by Varuzhanyan and colleagues aimed to investigate the significance of mitochondrial fusion in mouse spermatogenesis. They found that double mutants for MFN-1 and MFN-2 were unable to produce any sperm, indicating that mitochondrial fusion is necessary for proper spermatogenesis functioning [137]. In Drosophila melanogaster, a mutation in the mitofusin homolog (Marf) has been associated to male sterility, being essential for the maintenance of male germline stem cells [138]. Therefore, it is evident that mitochondrial fusion/fission is essential for the correct functioning of spermatogenesis and these mechanisms could pointed as a possible biomarker on mitochondrial fitness that can be correlated with spermatozoa health (Figure 3). Additionally, recent findings have highlighted the critical role of autophagy in post-meiotic spermatids for cellular remodelling [139]. Although excess mitochondria are removed during spermatogenesis, the involvement of mitophagy in maintaining mitochondrial quality in the male reproductive tract is still under intense debate and requires further understanding [26].

Throughout the male reproductive tract, a significant amount of energy is required to complete the complex processes involved in spermatogenesis and oocyte fertilization. The primary source of energy is derived from OXPHOS [140]. Sperm contains substrates such as glutamic acid (GLU) [141], pyruvate, and lactate that serve as potential energy sources [142]. GLU can be converted into α-ketoglutarate through reactions involving alanine aminotransferase (ALT) and aspartate aminotransferase (AST). In this process, pyruvate and oxaloacetate are transformed into alanine and aspartate, respectively, which contribute to OXPHOS [141]. Alternatively, pyruvate can be reduced to L-lactate by lactate dehydrogenase, generating NAD+ for glycolysis. Pyruvate can also enter the Krebs cycle to support OXPHOS [99]. LDH activity in the mitochondrial matrix of sperm was first discovered in rabbit epididymal spermatozoa and has since been reported in several species, including humans [143]. In humans, LDH-C4 is present in the sperm mitochondrial matrix and facilitates the conversion of lactate to pyruvate, thereby aiding in mitochondrial energy production [144]. Studies have shown that supplementation of sperm media with lactate and pyruvate improves mitochondrial function compared to media containing only glucose [145]. However, this finding has sparked debate over the preferred metabolic pathway for maintaining optimal sperm function [146]. Both oxidative and glycolytic energy metabolism pathways work in tandem to generate energy [145]. Despite the ongoing debate, the crucial question remains whether sperm mitochondria should operate at their maximum capacity, as increased mitochondrial activity leads to increased ROS production [145]. As previously mentioned, ROS are considered harmful by-products of mitochondrial metabolism. When the level of ROS exceeds the antioxidant defences of the cell, it leads to cell damage and oxidative stress [147]. However, mild oxidative stress is necessary for sperm functions such as fertilization, motility, and capacitation, despite causing damage to sperm structure and function [148]. For example, ROS can promote capacitation by regulating tyrosine phosphorylation through redox, which enhances the sperm’s ability to bind to the zona pellucida [149]. Research on stallion spermatozoa has associated increased ROS production with rapid mitochondrial activity in sperm [142]. In another study, higher ROS levels were observed in stallion spermatozoa with good freezability after cryopreservation, compared to those with poor freezability, indicating increased mitochondrial activity [150]. Therefore, while excessive ROS production can cause OS and impair sperm function, a moderate amount of ROS is required for proper sperm function. The optimal balance between ROS generation and antioxidant defence should be maintained to support healthy sperm function [145]. In conclusion, mitochondria play a vital role in male fertility, and their quality and proper functioning in sperm are maintained not only through biogenesis mechanisms, but also by balancing oxidative and glycolytic metabolic pathways and by balancing the overproduction and underproduction of ROS. The goal is to prevent cell damage, oxidative stress, and apoptosis, and to enable all phases of the male reproductive tract to function properly. Maintaining optimal mitochondrial function is essential for healthy sperm and successful fertilization. Therefore, future research should focus on understanding the precise mechanisms that regulate mitochondrial metabolism and identifying potential therapeutic targets to enhance mitochondrial function and improve male fertility.

## 6. Conclusions

Mitochondria are essential organelles for male fertility, and their correct functioning is critical for the success of the reproductive process. Mitochondria play an essential role in generating ATP, the energy required for the physiological mechanisms involved in spermatogenesis and fertilization. However, mitochondria also produce ROS, which can lead to cellular damage, oxidative stress, and apoptosis if not properly maintained. One of the crucial functions of mitochondria in sperm is to produce ATP, which is necessary for various sperm processes, including motility, hyperactivation, capacitation, and acrosome reaction. The correct functioning of mitochondria in sperm is directly related to sperm quality, and defects in mitochondrial function can lead to male infertility. Mitochondria are susceptible to damage from both internal and external factors. Internal factors include overproduction of ROS by leucocytes, immature germ cells, and defective spermatozoa, which can cause oxidative stress and damage mitochondria. External factors that can damage mitochondria include NCDs, such as obesity and diabetes, aging, and poor health habits such as lack of exercise and smoking. Mitochondria have quality control mechanisms to prevent malfunctions. One such mechanism is biogenesis, which ensures that only functional mitochondria are retained, and dysfunctional ones are eliminated. Metabolic pathways in sperm also play a crucial role in maintaining mitochondrial function, by balancing oxidative and glycolysis metabolic pathways and maintaining a physiological equilibrium in the production of ROS. Assessing mitochondrial functionality and quality control is crucial to understanding and managing male infertility. Measuring mitochondrial DNA quality and assessing mitochondrial function in sperm can provide valuable information for diagnosing and treating male infertility. Additionally, developing interventions that target the mitochondrial pathways involved in sperm function may lead to improved treatments for male infertility. In conclusion, mitochondria play a critical role in male fertility, and their correct functioning is essential for the success of the reproductive process. Maintaining a balance between ATP generation and ROS production is necessary to prevent damage to the sperm and maintain sperm quality. By understanding the mechanisms involved in mitochondrial function and quality control, we can develop effective interventions for the treatment of male infertility.

## Data Availability

Not applicable.
